# Low birth weight and reduced postnatal nutrition lead to cardiac dysfunction in piglets

**DOI:** 10.1093/jas/skad364

**Published:** 2023-10-25

**Authors:** Ashley C McPeek, Breanna Patton, Daniel A Columbus, T Dylan Olver, Lucas A Rodrigues, Jade M Sands, Lynn P Weber, David P Ferguson

**Affiliations:** Department of Kinesiology Michigan State University, East Lansing, MI, 48824, USA; Center for Health Sciences Interprofessional Education, Research, and Practice, University of Washington, Seattle, WA 98144, USA; Department of Veterinary Biomedical Sciences, University of Saskatchewan, Saskatoon, SK S7N 5B4, Canada; Department of Animal and Poultry Science, University of Saskatchewan, Saskatoon, SK S7N 5A8, Canada; Prairie Swine Centre, Inc., Saskatoon, SK S7H 5N9, Canada; Department of Veterinary Biomedical Sciences, University of Saskatchewan, Saskatoon, SK S7N 5B4, Canada; Department of Animal and Poultry Science, University of Saskatchewan, Saskatoon, SK S7N 5A8, Canada; Prairie Swine Centre, Inc., Saskatoon, SK S7H 5N9, Canada; Department of Animal and Poultry Science, University of Saskatchewan, Saskatoon, SK S7N 5A8, Canada; Prairie Swine Centre, Inc., Saskatoon, SK S7H 5N9, Canada; Department of Veterinary Biomedical Sciences, University of Saskatchewan, Saskatoon, SK S7N 5B4, Canada; Department of Kinesiology Michigan State University, East Lansing, MI, 48824, USA

**Keywords:** cardiovascular disease, growth restriction, low birth weight, porcine, undernutrition

## Abstract

Heart disease is the leading cause of death in humans and evidence suggests early life growth-restriction increases heart disease risk in adulthood. Therefore, this study sought to investigate the effects of low birth weight (LBW) and postnatal restricted nutrition (RN) on cardiac function in neonatal pigs. We hypothesized that LBW and RN would reduce cardiac function in pigs but this effect would be reversed with refeeding. To investigate this hypothesis, pigs born weighing <1.5 kg were assigned LBW, and pigs born >1.5 kg were assigned normal birth weight (NBW). Half the LBW and NBW pigs underwent ~25% total nutrient restriction via intermittent suckling (assigned RN) for the first 4 wk post-farrowing. The other half of piglets were allowed unrestricted suckling access to the sow (assigned NN). At 28 d of age (weaning), pigs were weaned and provided ad libitum access to a standard diet. Echocardiographic, vascular ultrasound, and blood pressure (BP) measurements were performed on day 28 and again on day 56 to assess cardiovascular structure and function. A full factorial three-way ANOVA (NN vs. RN, LBW vs. NBW, male vs. female) was performed. Key findings include reduced diastolic BP (*P* = 0.0401) and passive ventricular filling (*P* = 0.0062) in RN pigs at 28 d but this was reversed after refeeding. LBW piglets have reduced cardiac output index (*P* = 0.0037) and diastolic and systolic wall thickness (*P* = 0.0293 and *P* = 0.0472) at 56 d. Therefore, cardiac dysfunction from RN is recovered with adequate refeeding while LBW programs irreversible cardiac dysfunction despite proper refeeding in neonatal pigs.

## Teaser

Poor cardiac development is linked with low birth weight and nutrient intake in piglets. Nutrient-induced dysfunction, but not birth weight-related dysfunction, is reversed by refeeding.

## Introduction

Heart disease is the leading cause of death in adults, with 17 million deaths occurring per year (WHO, 2020). There are several known modifiable risk factors linked to the development of heart disease including sedentary lifestyle, smoking, and poor dietary nutrient intake (Hajar, 2017). However, more recently, studies have confirmed that restricted nutrition (RN) during either intrauterine growth or postnatal growth are risk factor for impaired cardiac development in human and animal models (Katsumata et al., 2000; Kaijser et al., 2008; Fouzas et al., 2014; Fabiansen et al., 2015; Ferguson et al., 2019, 2021; Visker et al., 2020). Globally, 160 million children under the age of five experience a poor nutritive environment leading to growth restriction (Onis and Branca, 2016; Blencowe et al., 2019). Retrospective cohort studies in humans have shown that low birth weight (LBW) is associated with an increased risk of cardiovascular disease in adulthood, which may be exacerbated by poor postnatal nutrition (Osmond and Barker, 2000; Lumey et al., 2007; Kaijser et al., 2008; Thornburg, 2015). Due to the ethical concerns and logistical difficulties in conducting controlled human/infant studies on growth restriction, translational animal models are necessary to determine physiological mechanisms and develop therapeutic countermeasures (Baker, 2008; Odle et al., 2014).

Rodents are commonly used to model human growth-restriction due to their accelerated lifespans, similar physiology, and genetic structure (Clarke, 2002; Bryda, 2013; Yue et al., 2014; Mitchell et al., 2015; Kõks et al., 2016). The literature has shown that growth-restricted mice have smaller hearts, impaired cardiac excitation-contraction mechanics, and reduced cardiac function which manifests as reduced functional capacity (Murça et al., 2012; Visker and Ferguson, 2018; Ferguson et al., 2019; Pendergrast et al., 2020). While this information is important for human health care, a mouse heart continues to develop postnatally while human hearts achieve terminal differentiation during gestation, which could limit translatability of mouse studies to humans. However, pigs are thought to be the most closely related to humans of any animal model other than primates (Baker, 2008) A pig’s heart shares more similarities in anatomy and physiology to a human heart and a pig’s cardiac morphogenesis is mostly completed by gestational day 42, which is similar to human development (Gabriel et al., 2021). These similarities make pigs an ideal preclinical model for translational relevance for human cardiovascular health. Therefore, the next step is to determine how early life growth restriction influences cardiac function as the pig ages and if refeeding an adequate diet could mitigate cardiac dysfunction. (Baker, 2008; Devaskar and Chu, 2016; Huting et al., 2019).

The aim of the present investigation was to determine the influence of birth weight and postnatal nutrient intake on cardiac function and structure (as measured by echocardiography) in neonatal pigs. The specific objectives were to (1) determine baseline cardiac function and vascular health of weanling piglets that were both naturally occurring low and normal birth weight (NBW)s at 28 d old after adequate or inadequate postnatal nutrition and (2) determine the same cardiac and vascular parameters in LBW and NBW piglets (post-weaning), who have been re-fed with proper nutrition for 4 wk (56 d old). We hypothesized that LBW and RN during the immediate postnatal period would result in poor cardiac development that would lead to cardiac dysfunction in piglets but would be reversed by proper refeeding post-weaning.

## Methods

All procedures contributing to this work complied with the ethical standards of the Canadian Council of Animal Care guidelines for the care and use of farm animals in research and were approved by the Animal Research Ethics Board of the University of Saskatchewan (AUP# 20190042).

### Animal housing and experimental design

The present investigation is a subset of data collected from a larger investigation by Rodrigues et al. (Rodrigues et al., 2020). Briefly, a nutritive model for LBW pigs was developed as follows: within 48 h after farrowing, piglets were cross-fostered, if required, between sows to standardize litter size to 12–14 piglets/litter. Piglets that were randomly born less than 1.5 kg at birth were considered LBW and greater than 1.5 kg were considered NBW, an identifying convention accepted in the established peer-reviewed literature (Beaulieu et al., 2010). All sows were treated identically and were provided the same commercial swine diet during both gestation and lactation (Rodrigues et al., 2020). Postnatal RN was induced in four piglets per litter (two LBW and two NBW) via intermittent suckling (Berkeveld et al., 2007; Kuller et al., 2007). Intermittent suckling was induced by isolating piglets from the sow for 6 h/d from 08:00 to 14:00 h from day 3 post-farrow until weaning at day 28. This results in a ~25% reduction in feed intake as published previously and estimated in the original study (Berkeveld et al., 2007; Rodrigues et al., 2020). (Berkeveld et al., 2007; Rodrigues et al., 2020). All other piglets were allowed unrestricted suckling access to the sow (normal nutrition [NN]). At the end of the suckling period (day 28), 32 piglets, eight per treatment group were randomly chosen for cardiac assessment (NBW NN *n* = 3 males, five females; LBW NN *n* = 4 males, four females; NBW RN *n* = 4 males, four females; LBW RN *n* = 4 males, four females). The remaining piglets were weaned onto a commercial nursery diet (Masterfeeds) that was formulated to meet nutrient requirements (Council, 2012) and provided ad libitum until 56 d old. Weaned piglets were housed in groups of 3–6/pen within their treatment group. After 4 wk (day 56), an additional 32 piglets were randomly chosen for cardiac assessment (NBW NN *n* = 4 males, 4 females; LBW NN *n* = 4 males, four females; NBW RN *n* = 4 males, four females; LBW RN *n* = 3 males, five females).

### Echocardiography and vascular ultrasonography

During the nutrient restriction and refeeding phases of the study, piglets were housed at the Prairie Swine Centre (Saskatoon, SK). At days 27 and 55, four piglets from each group were randomly selected, weighed, and transported to the Western College of Veterinary Medicine’s Animal Care Unit (Saskatoon, SK) where they were fasted for 24 h prior to echocardiography and ultrasonography measurements. Piglets were anesthetized with 5% isoflurane and maintained under 2% isoflurane during cardiac measurements. No premedication was used, and the palpebral reflex was assessed to ensure piglets were fully under anesthesia. As such, anesthesia maintenance was adjusted as needed for each individual pig based on real-time physiological parameters. Blood pressure (BP), heart rate (HR), and temperature were recorded throughout the procedure. BP was measured at least every 5 min using a high-definition oscillometric BP machine (VET HDO High-Definition Oscillometer, Babenhausen, Germany) on the right forelimb mid-length over the radial and ulnar bones. An average of readings taken during the 20-min sonographic exam for each pig was used to determine the systolic and diastolic pressures. HR was recorded from electrocardiography (ECG) and values reported were an average of all values throughout the procedure.

A SonoSite M Turbo Ultrasound System (Fujifilm Sonosite, Markham ON Canada) was used to obtain all echocardiography and Doppler images. Piglets were placed in left lateral recumbency to obtain two-chamber and four-chamber apical views. The Doppler cursor was placed directly above mitral valve. Using Color mode and Doppler mode in the five-chamber view Isovolumetric Relaxation Time (IVRT) and IVRT as a fraction of cardiac cycles was calculated. E and A Waves were obtained in a two-chamber view (Schwarz et al., 2019). Finally, piglets were flipped to right lateral recumbency to obtain a short axis view of the left ventricle (LV) at the level of the papillary muscles. M-mode in short axis view was used to obtain ventricular wall movement, calculate stroke volume, cardiac output, ejection fraction, and fractional shortening. M-mode was also used to determine ventricular wall thickness and left ventricular volumes (Geva and Velde, 2006). Free wall and interventricular wall movement was qualitatively evaluated based on a five-point scale, with lower scores representing little to no wall movement indicative of wall stiffness. All measurements were calculated and analyzed during the procedure.

The same software (Fujifilm SonoSite M Turbo Ultrasound System) was used to obtain all vascular ultrasonography images. However, there is no doppler analysis available on the Fujifilm SonoSite vascular probe, so a separate program was used for analysis of vascular images (Adobe Premiere Elements 2019). Piglets were placed back into left lateral recumbency, and the left forelimb was used for vascular analysis. The Doppler cursor was placed on the brachial artery proximal to branching. Vessel diameter was measured at baseline (VDt0) and 30 seconds post a 60-second occlusion (VDt90). Images of brachial artery at maximum dilation were pulled from ultrasound video clips using Adobe Premiere Elements 2019 and V_max_ for Doppler waves and average vessel diameter were analyzed with Image Pro 10.

BP was obtained using a high-definition small animal oscillometer BP cuff (VET HDO High-Definition Oscillometer, Babenhausen, Germany) placed over the brachial artery in the right forelimb and recorded every 5 min during the ultrasound examination. Readings over the course of the examination were averaged, with a minimum of three readings with good agreement utilized to establish diastolic and systolic pressures.

### Statistics

Statistical analysis was performed using JMP Pro v14.0 (SAS, Cary, NC). Data were evaluated for normality with Shapiro–Wilk’s Normality test and outliers were determined from Grubb’s Outlier test. Outliers were removed and if variables failed normality, a Log-transformation was used before running a full factorial three-way ANOVA with feed (NN vs. RN), birth weight (LBW vs. NBW), and sex (male vs female) as the variables at 28 and 56 d. Variables that were log-transformed included: cardiac output, cardiac output index, E-wave time integral, LV wall movement, free wall movement, VDt0 velocity, VDt90 velocity, flow-mediated dilation, A-wave velocity and time, IVRT as a fraction, VDT0, corrected EDV and corrected ESV. Echocardiography structural components were run with a covariate of body surface area (BSA) and functional components were run with a covariate of HR. BSA was calculated by 734 × (Body Weight^0.656) (Kelley et al., 1973). No BSA covariate was used on variables already corrected by echocardiogram software. All significant, multiple comparisons were then assessed with a Tukey’s HSD post hoc test. The alpha level was set at *P* < 0.05 for two-way interactions and *P* < 0.10 for three-way interactions. All data is expressed as mean ± SEM. Figures are condensed for clarity by showing only the significant main or interaction effect.

## Results

### Cardiac structure and function

**Figure 1.**
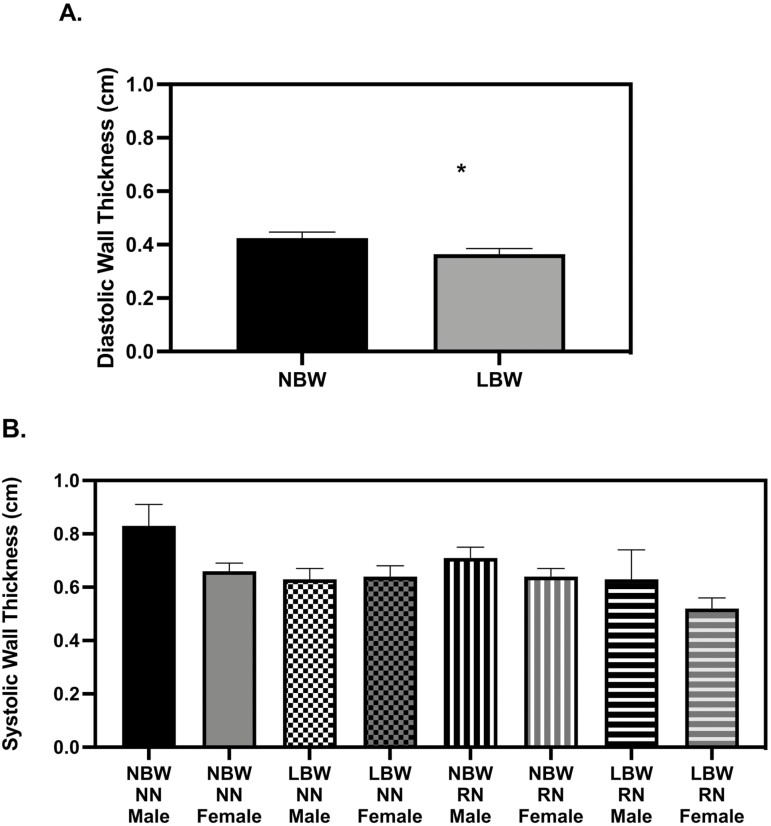
Left ventricle wall thickness at 28 d old. (A) low birth weight (LBW) pigs had thinner diastolic walls (*P* = 0.0492), (B) No differences existed in systolic wall thickness (*P* > 0.05). The echocardiography measures were analyzed with a 3-way full factorial ANOVA. Figures are condensed for clarity by showing only the significant main or interaction effect. Data are presented as mean ± SEM. Differences between groups (*P* < 0.05) are denoted by ‘*’. normal birth weight (NBW) NN *n* = 4 males, four females, LBW NN *n* = 4 males, four females NBW restricted nutrition (RN) *n* = 4 males, four females, and LBW RN *n* = 3 males, five females.

**Figure 2.**
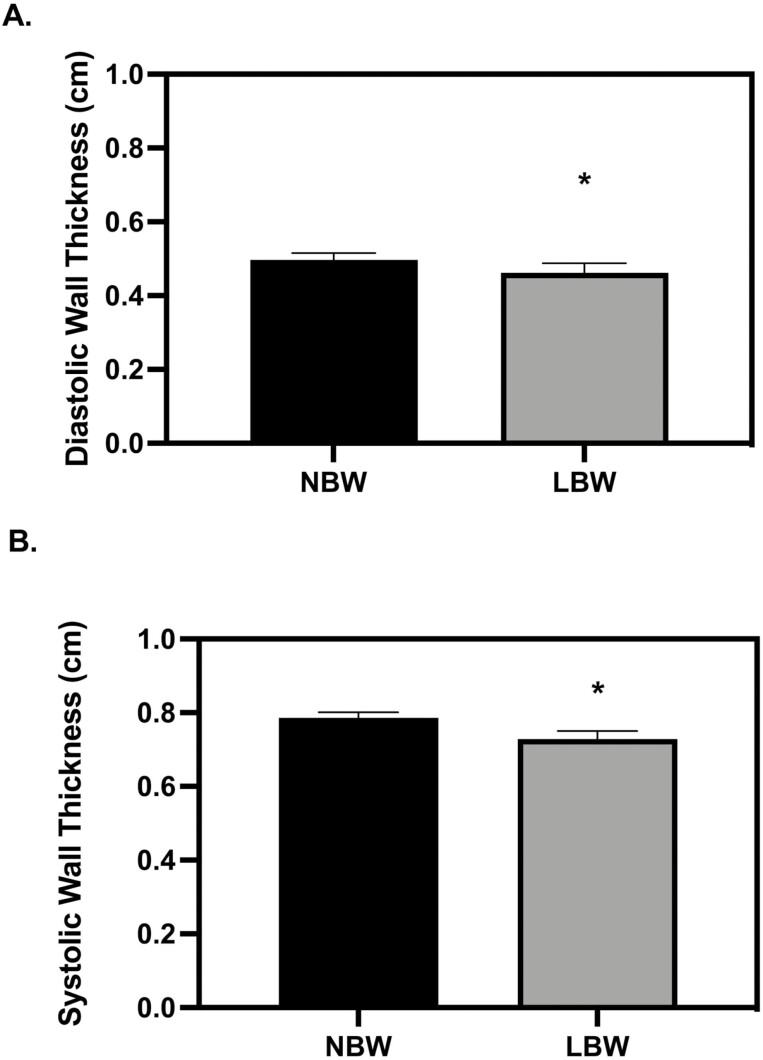
Left ventricle wall thickness at 56 d old. (A) low birth weight (LBW) pigs had thinner diastolic walls (*P* = 0.0293) and (B) thinner systolic walls as compared to normal birth weight (NBW) pigs (*P* = 0.0472). The echocardiography measures were analyzed with a 3-way full factorial ANOVA. Figures are condensed for clarity by showing only the significant main or interaction effect. Data are presented as mean ± SEM. Differences between groups (*P* < 0.05) are denoted by ‘*’. NBW NN *n* = 4 males, four females, LBW NN *n* = 4 males, four females NBW restricted nutrition (RN) *n* = 4 males, four females and LBW RN *n* = 3 males, five females.

All measured cardiac variables were significantly different between 28 and 56 d old, in line with typical growth and maturation data growth data published by Rodrigues et al., (2020). There were no significant differences in end-systolic volume (ESV) or end-diastolic volume (EDV) between birth weight or diet groups at 28 d (Table 1). ESV and EDV were not different between birth weights at 56 d when normalized by body mass, but a sex effect showed females had smaller ESV and EDV than males (Table 2, *P* = 0.0467, *P* = 0.0109). LBW pigs had reduced wall thickness during diastole at 28 d but differences in wall thickness during systole (Figure 1, *P* = 0.0492). At 56 d, LBW pigs had reduced wall thickness during diastole and systole as compared to NBW pigs ­(Figure 2, *P* = 0.0293 and *P* = 0.0472, respectively).

**Table 1.** Echocardiography and sonography measures at age 28 d^1^

Birth weight category	NBW		LBW		NBW		LBW		*P-*value						
Feeding	NN		NN		RN		RN								
Sex	M *n* = 3	F *n* = 5	M *n* = 4	F *n* = 4	M *n* = 4	F *n* = 4	M *n* = 4	F *n* = 4	N	BW	S	N*BW	N*S	BW*S	N*BW*S
ESV, mL	14.3 ± 0.8	11.0 ± 1.3	11.4 ± 1.8	11.5 ± 2.8	11.0 ± 1.6	14.5 ± 2.6	11.9 ± 2.6	8.6 ± 1.2	n.s.	0.085	n.s.	n.s.	n.s.	n.s.	n.s.
EDV, mL	30.8 ± 6.0	26.2 ± 1.9	22.9 ± 3.3	24.7 ± 3.5	23.8 ± 2.6	27.9 ± 2.3	21.0 ± 2.9	18.2 ± 1.4	0.070	n.s.	n.s.	n.s.	n.s.	n.s.	n.s.
EF, %	51.2 ± 6.7	58.1 ± 3.7*	50.3 ± 1.5	55.6 ± 5.4*	54.4 ± 5.1	48.9 ± 5.3*	46.0 ± 7.3	42.3 ± 3.6*	n.s.	n.s.	0.040	n.s.	n.s.	n.s.	n.s.
FS, %	25.3 ± 4.4	29.3 ± 2.4	24.3 ± 1.0	28.0 ± 3.4	27.1 ± 3.1	24.0 ± 3.0	22.3 ± 4.1	20.8 ± 2.4	n.s.	n.s.	n.s.	n.s.	n.s.	n.s.	n.s.
IVRT, ms	57.9 ± 6.1	52.5 ± 3.7	38.8 ± 7.3	54.7 ± 4.8	51.6 ± 8.2	51.6 ± 2.4	50.9 ± 6.3	31.3 ± 4.8	n.s.	0.073	n.s.	n.s.	n.s.	n.s.	n.s.

Sex effect denoted by *. No interaction effects were observed. End systolic volume (ESV), end-diastolic volume (EDV), ejection fraction (EF), fractional shortening (FS), isovolumic relaxation time (IVRT), birth weight (BW), normal birth weight (NBW), Low birth weight (LBW), normal nutrition (NN), restricted nutrition (RN), male (M), female (F), nutrition effect (N), not significant (n.s.), and sex (S).

^1^Values are presented as mean ± SEM. No interaction effects were observed.

**Table 2.** Echocardiography and sonography measures at age 56 d^1^

Birth weight category	NBW		LBW		NBW		LBW		*P-*value						
Feeding	NN		NN		RN		RN								
Sex	M *n* = 4	F *n* = 4	M *n* = 4	F *n* = 4	M *n* = 4	F *n* = 4	M *n* = 3	F *n* = 5	*N*	BW	S	N*BW	N*S	BW*S	N*BW*S
ESV, mL	1.82 ± 0.2	1.24 ± 0.1*	1.66 ± 0.2	1.22 ± 0.1*	1.34 ± 0.3	1.29 ± 0.1*	1.61 ± 0.1	1.26 ± 0.2*	n.s.	n.s.	0.047	n.s.	n.s.	n.s.	n.s.
EDV, mL	3.06 ± 0.4	2.65 ± 0.3*	2.95 ± 0.2	2.46 ± 0.1*	2.38 ± 0.3	2.57 ± 0.1*	2.9 ± 0.2	2.63 ± 0.4*	n.s.	n.s.	0.011	n.s.	n.s.	n.s.	n.s.
Systolic BP, mmHg	142.0 ± 15.1	129.0 ± 12.8	131.1 ± 2.5	150.7 ± 6.6	110.7 ± 3.9	138.3 ± 11.2	127.3 ± 6.4	151.4 ± 3.4	n.s.	n.s.	n.s.	n.s.	n.s.	n.s.	n.s.
Diastolic BP, mmHg	64.4 ± 10.7	61.6 ± 7.6	55.1 ± 3.8	69.0 ± 3.0	46.05 ± 7.9	53.6 ± 7.2	52.2 ± 5.4	63.0 ± 5.4	n.s.	n.s.	n.s.	n.s.	n.s.	n.s.	n.s.
EF, %	40.6 ± 3.5	51.0 ± 3.0*	44.0 ± 1.7	50.5 ± 2.9*	33.3 ± 3.1	49.7 ± 3.0*	44.6 ± 0.8	52.0 ± 2.2*	n.s.	n.s.	0.001	n.s.	n.s.	n.s.	n.s.
FS, %	19.5 ± 2.0	25.6 ± 2.1*	21.1 ± 1.1	24.9 ± 1.6*	16.3 ± 1.7	24.8 ± 1.9*	21.5 ± 0.5	26.1 ± 1.3*	n.s.	n.s.	0.001	n.s.	n.s.	n.s.	n.s.
IVRT, ms	63.1 ± 7.0	56.6 ± 4.5	59.4 ± 4.3	59.1 ± 6.7	41.9 ± 8.7	57.3 ± 5.4	59.6 ± 8.5	59.1 ± 1.4	n.s.	n.s.	n.s.	n.s.	n.s.	n.s.	n.s.
E/A Ratio	0.94 ± 0.18	0.95 ± 0.14	1.05 ± 0.12	0.80 ± 0.11	0.71 ± 0.20	0.98 ± 0.09	0.85 ± 0.22	0.74 ± 0.08	n.s.	n.s.	n.s.	n.s.	n.s.	n.s.	n.s.

Sex effect denoted by *. No interaction effects were observed. Blood pressure (BP), ejection fraction (EF), fractional shortening (FS), isovolumic relaxation time (IVRT), birth weight (BW), normal birth weight (NBW), low birth weight (LBW), normal nutrition (NN), restricted nutrition (RN), male (M), female (F), nutrition effect (N), not significant (n.s.), and sex (S).

^1^Values are presented as mean ± SEM. No interaction effects were observed.

**Figure 3.**
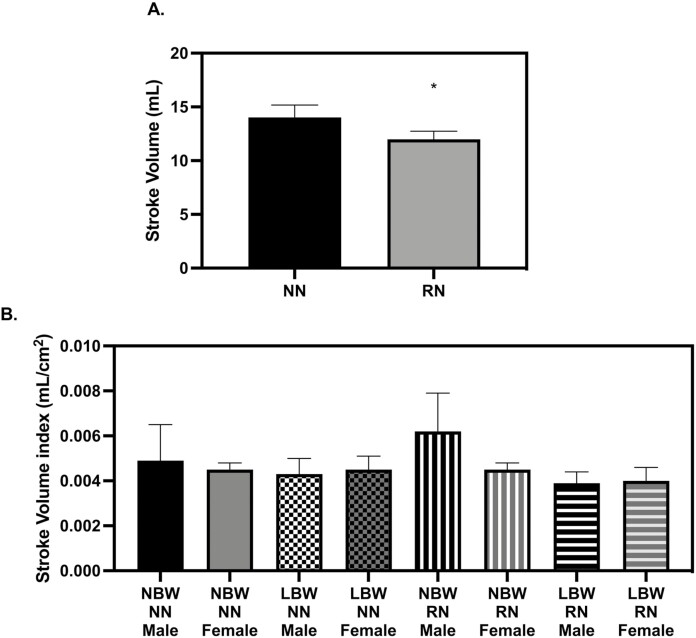
Stroke volume at 28 d old. (A) restricted nutrition (RN) pigs had smaller stroke volumes than NN pigs (*P* = 0.0495) (B) When normalized to body surface area, there were no differences in stroke volume (*P* > 0.05). The echocardiography measures were analyzed with a 3-way full factorial ANOVA. Figures are condensed for clarity by showing only the significant main or interaction effect. Data are presented as mean ± SEM. Differences between groups (*P* < 0.05) are denoted by ‘*’. normal birth weight (NBW) NN *n* = 4 males, four females, low birth weight (LBW) NN *n* = 4 males, 4 females NBW RN *n* = 4 males, four females and LBW RN *n* = 3 males, five females.

**Figure 4.**
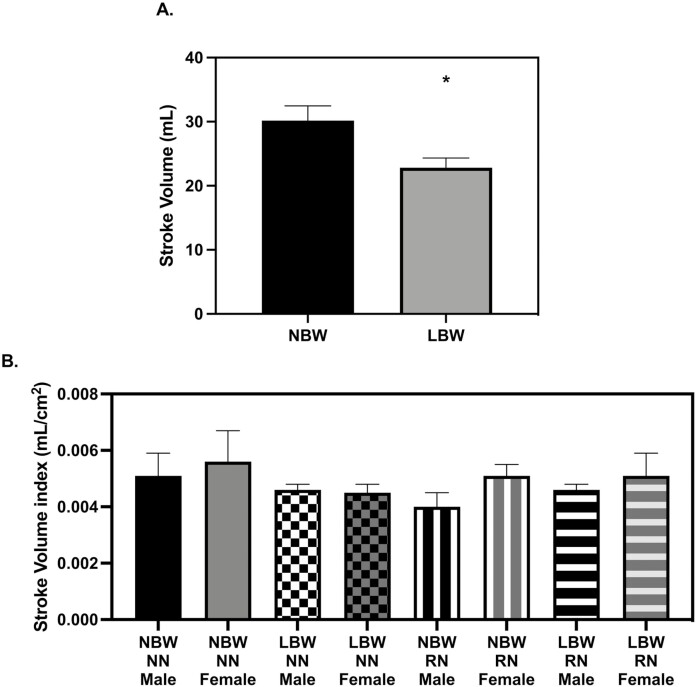
Stroke volume at 56 d old. (A) low birth weight (LBW) pigs had smaller stroke volumes than normal birth weight (NBW) pigs (*P* = 0.0397) (B) When normalized to body surface area, there were no differences in stroke volume (*P* > 0.05). The echocardiography measures were analyzed with a three-way full factorial ANOVA. Figures are condensed for clarity by showing only the significant main or interaction effect. Data are presented as mean ± SEM. Differences between groups (*P* < 0.05) are denoted by ‘*’. NBW NN *n* = 4 males, four females, LBW NN *n* = 4 males, four females NBW restricted nutrition (RN) *n* = 4 males, four females and LBW RN *n* = 3 males, five females.

**Figure 5.**
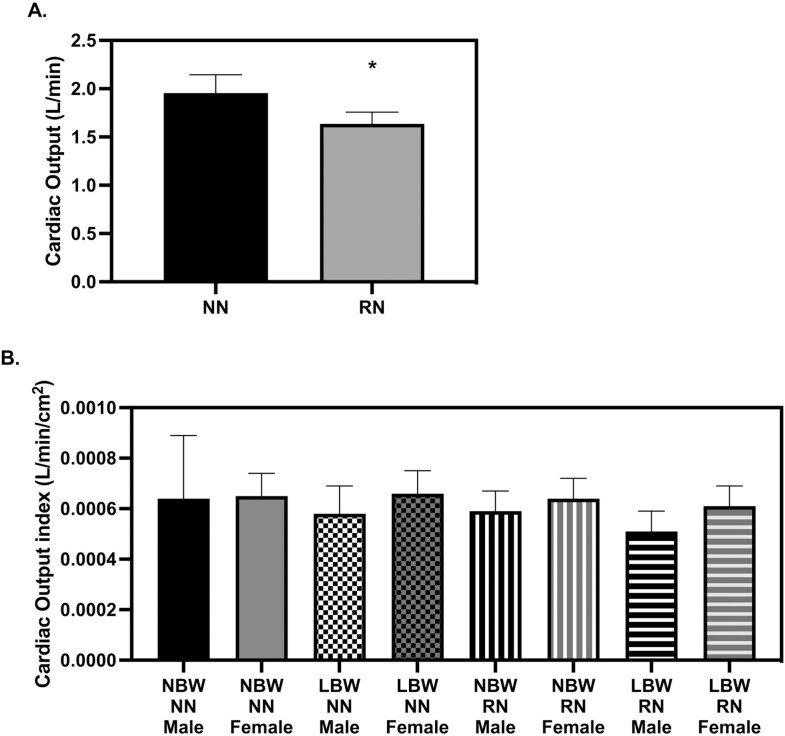
Cardiac output at 28 d old. (A) restricted nutrition (RN) pigs had smaller cardiac output than NN pigs (*P* = 0.0451) (B) When normalized to body surface area, there were no differences in cardiac output (*P* > 0.05). The echocardiography measures were analyzed with a 3-way full factorial ANOVA. Figures are condensed for clarity by showing only the significant main or interaction effect. Data is presented as mean ± SEM. Differences between groups (*P* < 0.05) are denoted by ‘*’. normal birth weight (NBW) NN *n* = 4 males, four females, low birth weight (LBW) NN *n* = 4 males, four females NBW RN *n* = 4 males, four females and LBW RN *n* = 3 males, five females.

**Figure 6.**
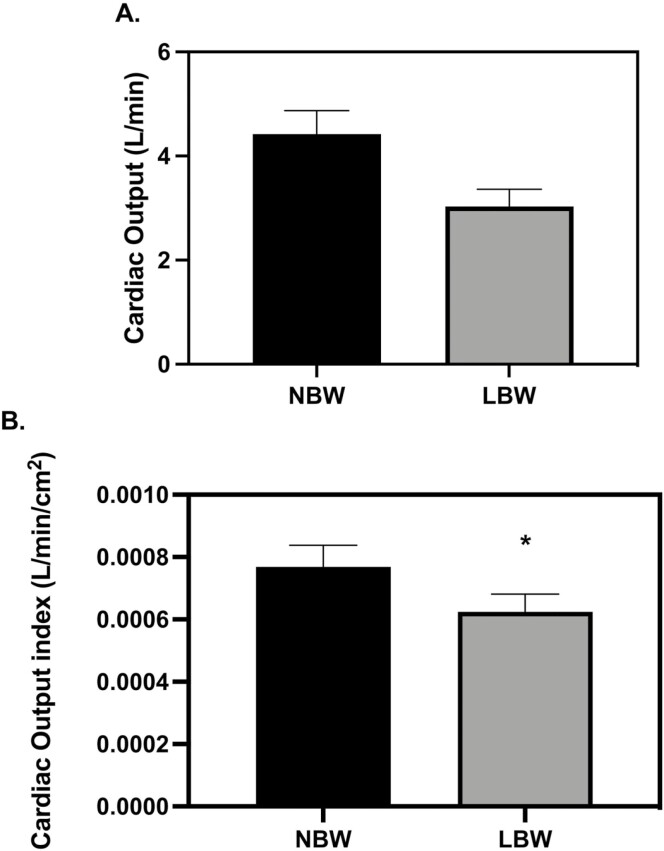
Cardiac output (Q) at 56 d old. (A) *A*bsolute Q was smaller in low birth weight (LBW) pigs compared to normal birth weight (NBW) pigs (*P* = 0.0037), (B) LBW pigs still had lower Q than NBW when Q was indexed to body surface area (*P* = 0.0337). The echocardiography measures were analyzed with a 3-way full factorial ANOVA. Figures are condensed for clarity by showing only the significant main or interaction effect. Data is presented as mean ± SEM. Differences between groups (*P* < 0.05) are denoted by ‘*’. NBW NN *n* = 4 males, four females, LBW NN *n* = 4 males, four females NBW restricted nutrition (RN) *n* = 4 males, four females and LBW RN *n* = 3 males, five females.

There was no difference in resting HR measures between groups. Stroke volume (SV) was lower in RN pigs when compared to NN pigs at 28 d old (Figure 3, *P* = 0.0495), but this effect was no longer present when indexed to BSA (Figure 3). At 56 d, SV was not smaller in RN pigs but instead, SV was lower in LBW when compared to NBW pigs (Figure 4, *P* = 0.0397). When indexed to BSA, differences in SV were not significant between groups (Svi, Figure 4). Absolute cardiac output (Q) was lower in RN pigs when compared to NN pigs at 28 d (Figure 5, *P* = 0.0451), but when Q was indexed to BSA, Q was not smaller in RN pigs (Qi, Figure 5) Absolute Q was not smaller in RN pigs as compared to NN pigs at 56 d, but was smaller in LBW pigs compared to NBW pigs at 56 d (Figure *P* = 0.0037). When Q was indexed to BSA (Qi), LBW pigs still had lower Q than NBW (Figure 6, *P* = 0.0337). Ejection fraction (EF) and fractional shortening (FS) were not affected by birthweight or diet at 28 or 56 d; however, female pigs had greater EF than males at 28 and 56 d (Tables 1 and 2, *P* = 0.0400 and *P* = 0.0007, respectively). FS was larger in females at 56 d (*P* = 0.0009, Table 2). There were no diet or birthweight effects on isovolumic relaxation time (IVRT) (Tables 1 and 2). Female pigs displayed a trend for greater free wall movement than males at both time points (*P* = 0.0677) but no differences in intraventricular (IV) wall movement were observed (Supplementary Table S1 and S2).

### Vascular structure and function

**Figure 7.**
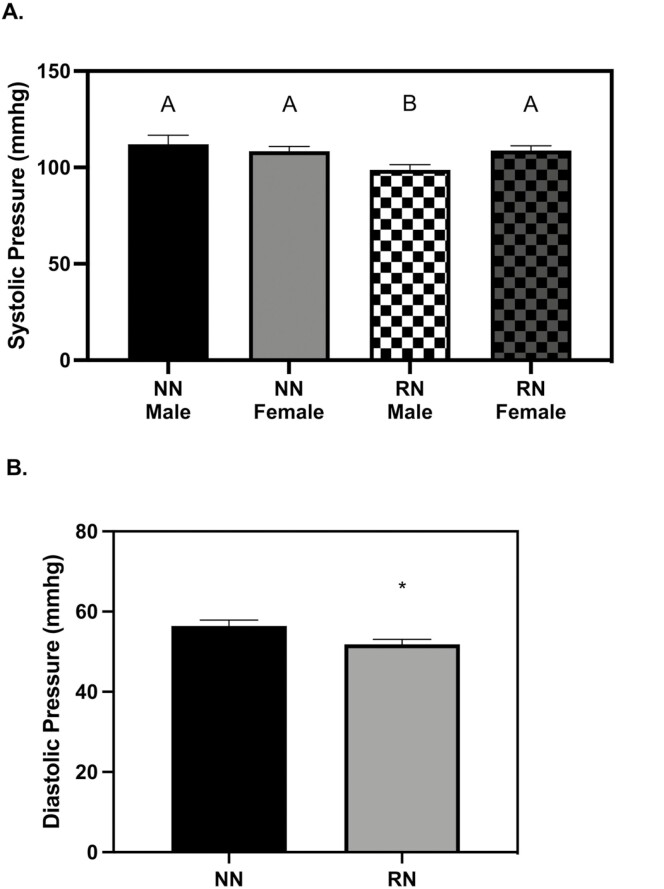
Blood pressure (BP) at 28 d old. (A) Male restricted nutrition (RN) pigs also had reduced systolic BP as compared to NN pigs (*P* = 0.0401) (B) All RN pigs had reduced diastolic BP (*P* = 0.0290). The vascular ultrasonography measures were analyzed with a 3-way full factorial ANOVA. Figures are condensed for clarity by showing only the significant main or interaction effect. Data are presented as mean ± SEM. Differences between groups (*P* < 0.05) are denoted by ‘*’. normal birth weight (NBW) NN *n* = 4 males, four females, low birth weight (LBW) NN *n* = 4 males, four females NBW RN *n* = 4 males, four females and low birth weight (LBW) RN *n* = 3 males, five females.

Systolic and diastolic BP were not different between the two-time points. At 28 d of age, all RN pigs had reduced diastolic BP (*P* = 0.0290), while the male RN pigs also had reduced systolic BP as compared to all other pigs (*P* = 0.0401, ­Figure 7). There were no diet or birth weight effects on systolic or diastolic BP at 56 d (Table 2).

**Figure 8.**
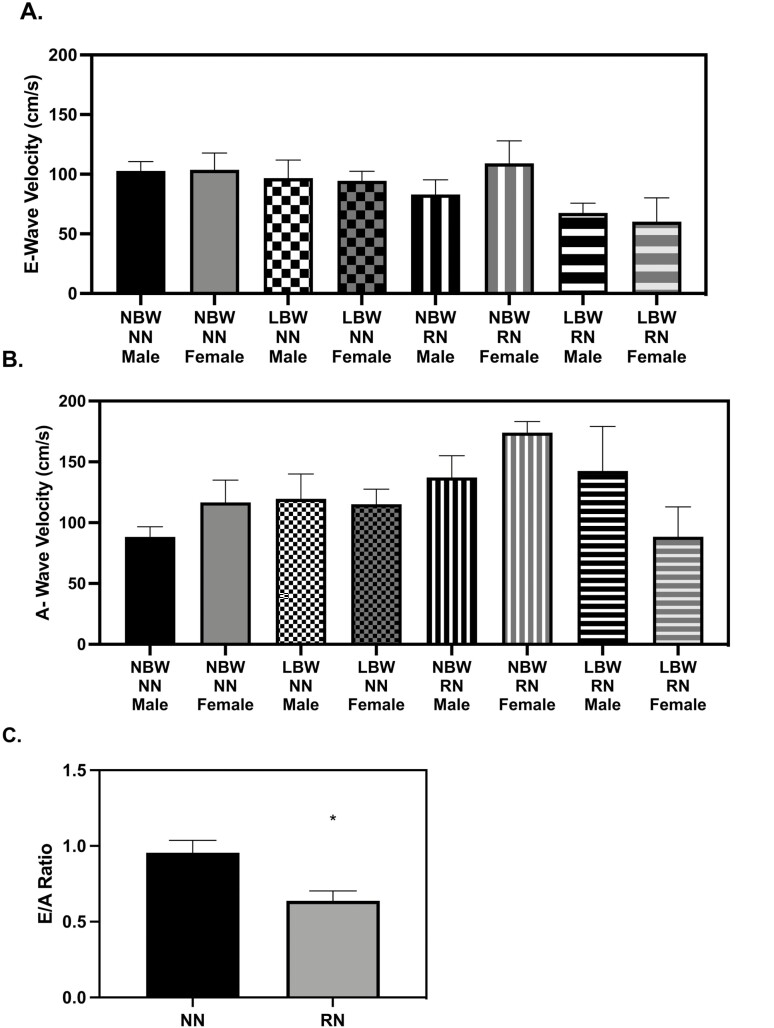
Diastolic function at 28 d old. (A) There were no birth weight or diet effects on E-wave (*P* > 0.05), (B) There were no birth weight or diet effects on A-wave peak velocity (*P* > 0.05). (C) restricted nutrition (RN) pigs had a decreased E/A ratio compared to NN at 28 d (*P* = 0.0062). The vascular ultrasonography measures were analyzed with a 3-way full factorial ANOVA. Figures are condensed for clarity by showing only the significant main or interaction effect. Data are presented as mean ± SEM. Differences between groups (*P* < 0.05) are denoted by ‘*’. normal birth weight (NBW) NN *n* = 4 males, four females, low birth weight (LBW) NN *n* = 4 males, four females NBW RN *n* = 4 males, four females and LBW RN *n* = 3 males, five females.

There were no age effects on vascular function variables between the two-time points. At 28 d old, there were no birth weight or diet effects on E-wave or A-wave peak velocity ­(Figure 8). RN had a decreased E/A ratio compared to NN at 28 d (Figure 8, *P* = 0.0062). RN pigs no longer had a reduced E/A ratio as compared to NN pigs at 56 d (Table 2). E-wave and A-wave velocity time integrals were not different between diet, birth weight, or sex groups at either time point (Supplementary Table S1 and S2).

No diet or birth weight effects were observed on flow-mediated dilation at either 28 or 56 d (Supplementary Table S1 and S2). At 28 d LBW pigs had smaller brachial artery diameter and vessel peak blood velocity time integral at baseline but no differences at t90 (Supplementary Table S1). At 56 d LBW pigs had smaller brachial artery vessel diameter at baseline and t90 than NBWpigs (Supplementary Table S2).

## Discussion

LBW has been linked to poor infant cardiac development and is a known risk factor for developing cardiac disease in adulthood (Vos et al., 2006; Thornburg, 2015; Huang et al., 2022) due to altered cardiac morphology (Fouzas et al., 2014). Additionally, poor cardiac health in livestock could hinder pork production if the pigs die before reaching the market. Thus, the issue of poor cardiac health is detrimental to human health and longevity, as well as agricultural production. The present investigation examined the effects of LBW and RN on cardiac structure and function in preweaning and re-fed piglets. The most important findings were (1) RN pigs had transient reduced cardiac function (E/A ratio and DBP) at 28 d old that was reversed once refeeding occurred and (2) LBW pigs presented with permanent cardiac dysfunction at 56 d (reduced LV volume, wall thickness, and Q) that were not recovered by refeeding.

From the original study by Rodrigues et al. (Rodrigues et al., 2020), LBW pigs maintained a smaller body mass than NBW pigs throughout the study, indicating a permanent stunting of growth in LBW pigs, which is associated with increased disease risk (Rodrigues et al., 2020). In contrast, the RN pigs were smaller than NN at 28 d old but were not different at 56 d, likely due to catch-up growth, defined as a compensatory accelerated growth after a period of growth inhibition, during the refeeding phase. Furthermore, Rodrigues et al. (Rodrigues et al., 2020) demonstrated reduced heart weight in both LBW and RN pigs at 28 d. However, at 56 d the RN pigs' heart mass increased, indicating cardiac-specific growth, while the LBW pigs still had a smaller heart weight than NBW (Rodrigues et al., 2020).

The lack of differences when LV volumes were normalized to body mass highlights the size of the heart was appropriate for the smaller LBW pigs. However, diastolic wall thickness was reduced in LBW pigs which agrees with earlier growth-restricted mice studies demonstrating reduced wall thickness and chamber volumes due to decreases in cardiomyocyte size and nucleation (Cohen et al., 2016; Ferguson et al., 2019). The reduction in wall thickness in LBW pigs is likely from smaller cardiomyocytes and is indicative of LV dilation to compensate for weaker cardiac muscles and to prevent a decrease in systolic function (Murça et al., 2012; Japp et al., 2016). LV dilation is a thinning of the ventricular walls and enlargement of the LV chamber, which is a well-recognized precursor to cardiac dysfunction and heart failure (Vasan et al., 1997). Since the pigs in this study are still juvenile, it is possible the LBW pigs will experience worsening cardiac function with an increased risk of heart failure in adulthood, due to morphological changes in the heart from early life. Further evidence for worsening cardiac function with aging comes from a recent study by Wellington et al. that reported impaired glucose tolerance in LBW piglets (Wellington et al., 2022). Wellington’s study suggests LBW pigs rely on fatty acid oxidation for energy due to an inability to metabolically switch to glucose metabolism due to growth restriction (Wellington et al., 2022). If true, this would support the current study where the heart is able to function seemingly normal in early life (as the heart relies primarily on fatty acid oxidation) but, with aging, glucose intolerance would disrupt cardiac function (Karwi et al., 2018).

There was no difference in resting HR between groups ensuring differences were due to interventions and not due to differences in contraction rates or anesthesia effects (Conrad et al., 1982; Lang et al., 2015; Wang et al., 2016). LBW pigs had reduced Qi at 56 d old, indicating reduced cardiac function as they aged. Previous literature is divided on what changes early life growth restriction have on systolic function and when they occur. Several studies have found LBW increases IVCT and FS but reduces SV to maintain a normal Q with a higher HR (Cohen et al., 2016; Ferguson et al., 2019), while others have reported no change at all in systolic parameters at rest (Shoukry et al., 1986; Fabiansen et al., 2015). Interestingly, the LBW pigs in this study did not show any changes in IVRT, FS, or SV and as such presented only with a decrease in Qi. Considering an anesthetic effect on HR, the reduction in Qi is likely related to the signs of LV dilation in LBW pigs, as a weaker dilated LV will not be able to pump efficiently and can get worse with aging (Akasheva et al., 2015). Thus, it is important to note the early age (56 d) at which the pigs in the current study were examined and the possibility of worsening cardiac dysfunction with maturity.

In contrast to the growth-restricted pigs in the current study, other animal models and human studies of LBW and RN have reported increased IVRT indicating ventricular stiffness and reduced diastolic function in adulthood (Fouzas et al., 2014; Fabiansen et al., 2015; Ferguson et al., 2019; Pendergrast et al., 2020; Visker et al., 2020). Since our study did not allow the pigs to age into adulthood, this may be one reason why IVRT was preserved in our pigs. Additionally, the use of different models to induce LBW and the difference in nutrition restriction methodologies (total vs. macronutrient restriction) can induce different physiological responses (Cohen et al., 2016; Dai et al., 2021). It is also possible that IVRT is not altered at rest, but only when the heart is under exertional stress (i.e., physical stress, pharmaceutical stress) is IVRT prolonged (Schmitz et al., 2004). Since our study only investigated parameters at rest, we cannot exclude the possibility that IVRT would be prolonged during exertion. Although IVRT can indicate cardiac stiffness and dysfunction, the E/A ratio is also an important indicator of impaired filling and diastolic dysfunction (Kossaify and Nasr, 2019). Thus, the overall decrease in the E/A ratio in all RN pigs, indicated poor passive ventricular filling and diastolic dysfunction (E/A < 0.8) (Kuznetsova et al., 2009; Cohen et al., 2016). Importantly, this did not persist at 56-d-old indicating adequate refeeding during a growth period was able to reverse cardiac dysfunction in RN pigs. It is also possible aging itself leads to growth in the pigs as 28 to 56 d is a post-weaning growth period. Despite not having a group continually RN, evidence in other animals suggests groups that are restricted longer (throughout life) than the current study do not recover from cardiac dysfunction as a result of aging (Marshall et al., 2022). It is likely during the refeeding period, RN pigs experienced normal developmental growth as the RN pigs’ body mass and heart mass were no longer smaller than the NN pigs at 56. Thus, refeeding during a period of growth was able to improve the E/A ratio through increased body mass and heart development and maturation (Harada et al., 1996).

In contrast to the growth-restricted pigs in the current study, other animal models and human studies of LBW and RN have reported increased IVRT indicating ventricular stiffness and reduced diastolic function in adulthood (Fouzas et al., 2014; Fabiansen et al., 2015; Ferguson et al., 2019; Pendergrast et al., 2020; Visker et al., 2020). Since our study did not allow the pigs to age into adulthood, this may be one reason why IVRT was preserved in our pigs. Additionally, the use of different models to induce LBW and the difference in nutrition restriction methodologies (total versus macronutrient restriction) can induce different physiological responses (Cohen et al., 2016; Dai et al., 2021). It is also possible that IVRT is not altered at rest, but only when the heart is under exertional stress (i.e., physical stress, pharmaceutical stress) is IVRT prolonged (Schmitz et al., 2004). Since our study only investigated parameters at rest, we cannot exclude the possibility that IVRT would be prolonged during exertion. Although IVRT can indicate cardiac stiffness and dysfunction, the E/A ratio is also an important indicator of impaired filling and diastolic dysfunction (Kossaify and Nasr, 2019). Thus, the overall decrease in the E/A ratio in all RN pigs, indicated poor passive ventricular filling and diastolic ­dysfunction (E/A < 0.8) (Kuznetsova et al., 2009; Cohen et al., 2016). Importantly, this did not persist at 56 d indicating adequate refeeding during a growth period was able to reverse cardiac dysfunction in RN pigs. During refeeding RN pigs experienced catch-up normal developmental growth as the RN pigs’ body mass and heart mass were the same size as NN pigs at 56 d. Thus, refeeding during a period of growth was able to improve the E/A ratio through catch-up growth, increased body mass, and heart development (Harada et al., 1996).

It is necessary to point out the value of the pig model in cardiovascular research. Pigs share many characteristics with humans not only in anatomy but also in physiology related to excitation-contraction coupling, myofilament composition, and contractile and relaxation kinetics. Pigs even respond to stressors (i.e., exercise) similarly to humans. Thus, the appropriate use of pig models has the potential to accelerate translation of data obtained to human infants and adults. Additionally, the pig is an agriculturally important livestock species, of which the pork industry has found a leading cause of deficits in pork production (i.e., pig deaths) are due to preexisting cardiac abnormalities. LBW has increased in pig offspring due to larger litter sizes and it is important to understand how nutrition effects each aspect of pig development. The current investigation not only adds evidence that pigs are a good model for translational studies but also contributes novel information for the pork production industry to focus on early feeding strategies and interventions (Shen et al., 2012; Heo et al., 2013).

### Limitations

As with many translational animal model studies, there are limitations to the study execution. Although a pig’s ­cardiovascular system is very similar to humans, it is still not a human system. Particularly, a pig’s cardiac growth does not allometrically scale with overall growth which leads to sudden cardiac death in pigs (Essen et al., 2018). However, this is an issue in larger pigs and is unlikely to effect early-life studies. This study was designed to have medium effect size with beta of 0.80 and alpha of 0.05, which is adequate for agricultural body mass studies (Rodrigues et al., 2020). Since this is a post hoc analysis of the previous study, we were adequately powered to achieve a medium effect size (Faul et al., 2007, 2009). To achieve a small effect size, 22 pigs per group would be needed; however, because of the expense of raising pigs, the current investigation adequately provides evidence for trends in cardiac function and warrants further research to continue. Due to the invasive nature of the larger study (Rodrigues et al., 2020), the pigs measured on day 28 are not the same pigs measured on day 56. BP measurements could be under or over-estimated in this study based on cuff size as cuff was fit based on closest cuff to fit 40% of limb circumference. Although some of our results conflict with previous literature, these differences are likely due to the methods of undernutrition and the LBW cutoff. Our study included pigs weighing ≤1.5 kg as LBW while other studies used ≤1.2 kg for LBW, but this was based on characteristics of the offspring population and previously established birth weight categories (Beaulieu et al., 2010). It is important to note that all our cardiac measures were taken under anesthesia and at rest, and some growth-restricted literature has shown normal cardiac function at rest but pathological dysfunction under stress (Drenckhahn et al., 2015; Visker and Ferguson, 2018). Thus, future studies should aim to examine cardiac function under stress as well.

## Conclusion

Our findings support the hypothesis that both LBW and RN offspring are linked to poor cardiac development, albeit with different effects for each insult. Poor cardiac development linked to LBW appears to be permanent as refeeding was not able to reverse cardiac dysfunction in the pigs at 56 d. Meanwhile, poor cardiac development is transient in RN pigs as refeeding was able to reverse cardiac dysfunction by 56 d. More research is needed to understand why diastolic BP and passive filling are most affected by LBW in early life by investigating cardiac structural (i.e., fibrosis) and functional changes (i.e., excessive sympathetic tone).

## Supplementary Material

skad364_suppl_Supplementary_Table_S1-S2

## Data Availability

Data is available upon reasonable request to the corresponding author.

## Abbreviations

BP: blood pressure
BSA: body surface area
ECG: electrocardiogram
EF: ejection fraction
EDV: end diastolic volume
ESV: end systolic volume
FS: Fractional Shortening
FMD: flow-mediated dilation
HR: heart rate
IVRT: isovolumic relaxation time
IV: interventricular
LBW: low birth weight
NBW: normal birth weight
NN: normal nutrition
Q: cardiac output
Qi: cardiac output index
RN: restricted nutrition
SV: stroke volume
VDt0: vessel diameter at baseline
VDt90: vessel diameter 90s post occlusion
