# Supplementary material for: Low birth weight and reduced postnatal nutrition lead to cardiac dysfunction in piglets
Source: J Anim Sci. 2023 Oct 25;101:skad364. doi: 10.1093/jas/skad364 (PMC10656296; doi:10.1093/jas/skad364)
Supplement: skad364_suppl_Supplementary_Table_S1-S2 [file skad364_suppl_supplementary_table_s1-s2.docx]

**Supplemental Table S1. Echocardiography and sonography measures at age 28 d in LBW or NBW pigs given either NN or RN pre-weaning^1^**

| **Birth Weight** | **NBW** | | **LBW** | | **NBW** | | **LBW** | | **p value** | | | | | | |
| --- | --- | --- | --- | --- | --- | --- | --- | --- | --- | --- | --- | --- | --- | --- | --- |
| **Nutrition** | **NN** | | **NN** | | **RN** | | **RN** | |  |  |  |  |  |  |  |
| **Sex** | **M**  n=3 | **F**  n=5 | **M**  n=4 | **F**  n=4 | **M**  n=4 | **F**  n=4 | **M**  n=4 | **F**  n=4 | **N** | **BW** | **S** | **N*BW** | **N*S** | **BW*S** | **N*BW*S** |
| IV wall movement | 2.67 ± 1.53 | 3.25 ± 0.96 | 2.25 ± 0.5 | 2.75 ± 0.96 | 2.75 ± 0.96 | 3.0 ± 1.63 | 3.25 ± 1.26 | 3.0 ± 0.82 | n.s. | n.s. | n.s. | n.s. | n.s. | n.s. | n.s. |
| Free wall movement | 3.67 ± 0.58 | 3.75 ± 0.96 | 2.75 ± 0.96 | 3.75 ± 1.26 | 3.0 ± 0.0 | 3.25 ± 0.96 | 3.25 ± 1.5 | 2.75 ± 1.71 | n.s. | n.s. | n.s. | n.s. | n.s. | n.s. | n.s. |
| E-wave velocity time integral (cm) | 6.92 ± 0.93 | 7.65 ± 0.92 | 4.72 ± .77 | 8.42 ± 0.93 | 4.37 ± 0.41 | 7.10 ± 1.06 | 5.05 ± 1.27 | 3.24 ± 1.86 | n.s. | n.s. | n.s. | n.s. | n.s. | n.s. | n.s. |
| A-wave velocity Time Integral (cm) | 3.72 ± 1.61 | 3.68 ± 0.71 | 3.88 ± 1.49 | 6.87 ± 3.29 | 4.07 ± 1.51 | 4.86 ± 0.67 | 9.03 ± 5.93 | 3.94 ± 0.91 | n.s. | n.s. | n.s. | n.s. | n.s. | n.s. | n.s. |
| FMD (%) | -5.92 ± 2.9 | 8.38 ± 5.05 | 17.93 ± 14.19 | -0.92 ± 2.97 | 7.23 ± 1.76 | -1.69 ± 8.57 | -1.83 ± 5.41 | 10.07 ± 7.28 | n.s. | n.s. | n.s. | n.s. | n.s. | n.s. | n.s. |
| Brachial artery vessel diameter t0 (cm) | 0.18 ± 0.01 | 0.17 ± 0.01 | 0.17 ± 0.01 | 0.17 ± 0.01 | 0.18 ± 0.01 | 0.2 ± 0.01 | 0.18 ± 0.01 | 0.13 ± 0.01 | n.s. | 0.015 | n.s. | n.s. | n.s. | n.s. | n.s. |
| Brachial artery vessel diameter t90 (cm) | 0.17 ± 0.01 | 0.19 ± 0.01 | 0.2 ± 0.02 | 0.17 ± 0.01 | 0.19 ± 0.01 | 0.19 ± 0.02 | 0.18 ± 0.02 | 0.14 ± 0.01 | n.s. | n.s. | n.s. | n.s. | n.s. | n.s. | n.s. |
| Vessel peak blood velocity t0 (cm/s) | 15.17 ± 3.99 | 26.13 ± 11.49 | 9.78 ± 3.95 | 28.95 ± 4.06 | 22.25 ± 2.51 | 25.88 ± 10.48 | 14.93 ± 3.15 | 13.76 ± 6.39 | n.s. | 0.045 | n.s. | n.s. | n.s. | n.s. | n.s. |
| Vessel peak blood velocity t90 (cm/s) | 17.83 ± 6.35 | 38.83 ± 6.76 | 19.58 ± 6.44 | 28.35 ± 4.02 | 19.83 ± 6.41 | 25.1 ± 5.9 | 17.33 ± 2.62 | 24.52 ± 7.8 | n.s. | n.s. | n.s. | n.s. | n.s. | n.s. | n.s. |

BW, birth weight; F, female; FMD, flow mediated dilation; IV, interventricular; LBW, low birth weight; M, male; N, nutrition effect; n.s., not significant; NBW, normal birth weight; NN, normal nutrition; RN,restricted nutrition; S, sex effect; t0, time 0; t90, time 90 s

^1^Values are presented as mean ± SEM. No interaction effects were observed.

**Supplemental Table S2. Echocardiography and sonography measures at age 56 d in LBW or NBW pigs given either NN or RN pre-weaning^1^**

| **Birth Weight** | **NBW** | | **LBW** | | **NBW** | | **LBW** | | **p value** | | | | | | |
| --- | --- | --- | --- | --- | --- | --- | --- | --- | --- | --- | --- | --- | --- | --- | --- |
| **Nutrition** | **NN** | | **NN** | | **RN** | | **RN** | |  |  |  |  |  |  |  |
| **Sex** | **M**  n=3 | **F**  n=5 | **M**  n=4 | **F**  n=4 | **M**  n=4 | **F**  n=4 | **M**  n=4 | **F**  n=4 | **N** | **BW** | **S** | **N*BW** | **N*S** | **BW*S** | **N*BW*S** |
| IV wall movement | 2.5 ± 0.58 | 3.5 ± 1.30 | 3.25 ± 1.26 | 3.25 ± 1.26 | 3.67 ± 1.53 | 2.6 ± 0.55 | 2.5 ± 0.58 | 4.0 ± 0.82 | n.s. | n.s. | n.s. | n.s. | n.s. | n.s. | n.s. |
| Free wall movement | 2.0 ± 0.82 | 3.5 ± 0.58 | 3.0 ± 0.82 | 3.25 ± 1.26 | 2.33 ± 0.58 | 3.8 ± 1.10 | 2.5 ± 1.30 | 3.25 ± 0.96 | n.s. | n.s. | n.s. | n.s. | n.s. | n.s. | n.s. |
| E-wave velocity time integral (cm) | 4.74 ± 0.61 | 3.96 ± 0.3 | 4.14 ± 0.78 | 5.31 ± 1.09 | 4.1 ± 1.23 | 5.7 ± 0.91 | 4.5 ±1.23 | 4.47 ± 0.78 | n.s. | n.s. | n.s. | n.s. | n.s. | n.s. | n.s. |
| A-wave velocity Time Integral (cm) | 3.84 ± 0.42 | 4.05 ± 0.48 | 4.79 ± 0.5 | 5.45 ± 0.81 | 3.62 ± 0.69 | 4.38 ± 0.72 | 3.74 ± 0.75 | 4.97 ± 0.65 | n.s. | n.s. | n.s. | n.s. | n.s. | n.s. | n.s. |
| FMD (%) | -0.19 ± 1.48 | 7.41 ± 1.22 | 2.18 ± 5.65 | 5.3 ± 5.64 | -2.5 ± 3.33 | -2.31 ± 1.37 | 3.81 ± 3.7 | -1.38 ± 3.13 | n.s. | n.s. | n.s. | n.s. | n.s. | n.s. | n.s. |
| Brachial artery vessel diameter t0 (cm) | 0.26 ± 0.02 | 0.24 ± 0.01 | 0.19 ± 0.01 | 0.2 ± 0.01 | 0.17 ± 0.02 | 0.23 ± 0 | 0.21 ± 0 | 0.23 ± 0.01 | n.s. | **0.009** | n.s. | n.s. | n.s. | n.s. | n.s. |
| Brachial artery vessel diameter t90 (cm) | 0.26 ± 0.02 | 0.26 ± 0.01 | 0.19 ± 0^ | 0.21 ± 0.01^ | 0.16 ± 0.02 | 0.23 ± 0.01 | 0.21 ± 0^ | 0.23 ± 0.02^ | n.s. | **0.005** | n.s. | n.s. | n.s. | n.s. | n.s. |
| Vessel peak blood velocity t0 (cm/s) | 27.1 ± 7.97 | 10.8 ± 3.06 | 13.55 ± 3.95 | 21.68 ± 5.87 | 23.58 ± 6.89 | 27.88 ± 8.68 | 12.25 ± 4.58 | 14.28 ± 8.7 | n.s. | n.s. | n.s. | n.s. | n.s. | n.s. | n.s. |
| Vessel peak blood velocity t90 (cm/s) | 29.78 ± 4.01 | 22.3 ± 7.3 | 23.33 ± 5.88 | 21.38 ± 7.79 | 21.73 ±7.59 | 24.12 ± 6.47 | 23.55 ± 7.08 | 16.23 ± 5.65 | n.s. | n.s. | n.s. | n.s. | n.s. | n.s. | n.s. |

BW, birth weight; F, female; FMD, flow mediated dilation; IV, interventricular; LBW, low birth weight; M, male; N, nutrition effect; n.s., not significant; NBW, normal birth weight; NN, normal nutrition; RN,restricted nutrition; S, sex effect; t0, time 0; t90, time 90 s

^1^Values are presented as mean ± SEM. No interaction effects were observed.
